# Supplementary material for: Population Subdivision and Migration Assessment of Mangalica Pig Breeds Based on Pedigree Analysis
Source: Animals (Basel). 2024 Feb 19;14(4):653. doi: 10.3390/ani14040653 (PMC10886115; doi:10.3390/ani14040653)
Supplement: Supplementary file 1 [file animals-14-00653-s001.zip › animals-2789879-supplementary.pdf]

animals

MDPI

Supplementary Materials

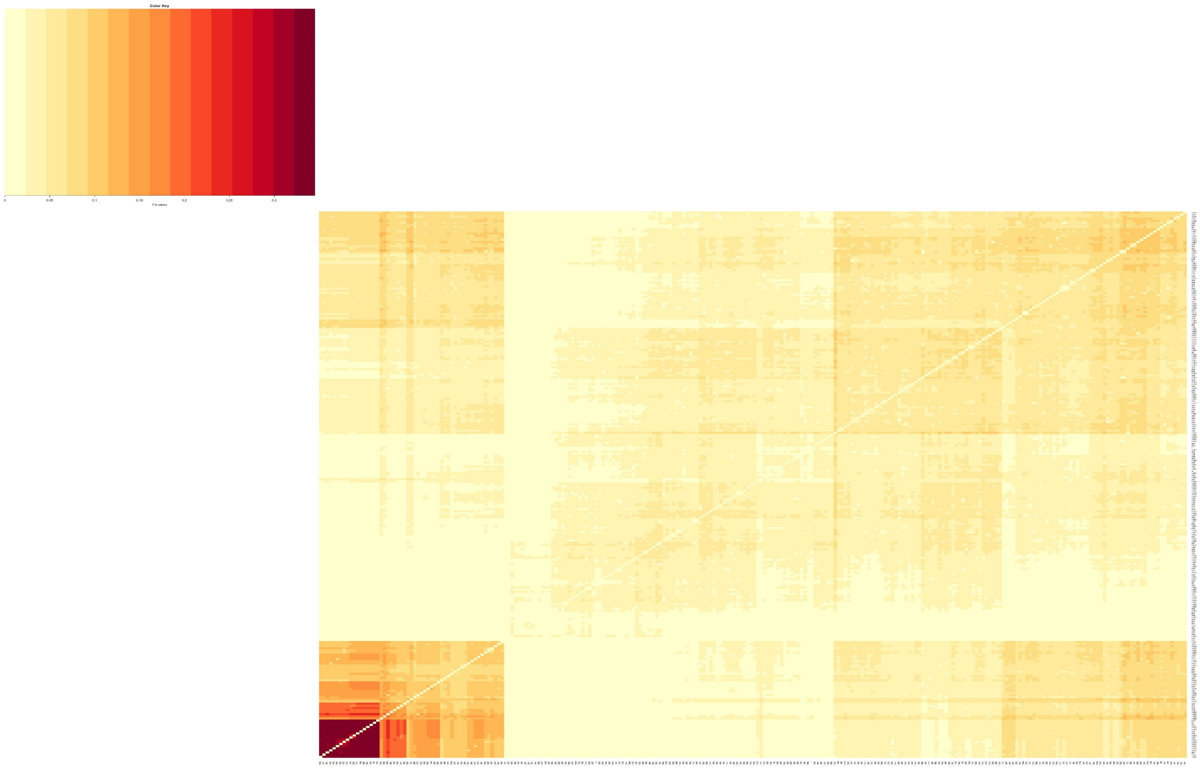

Figure S1. Heatmap based on pairwise *F*$_{ST}$ coefficients between the herds of Blonde Mangalica breed. The color key legend ranging from yellowish to dark red represents the scale of *F*$_{ST}$ coefficients from zero to onward.The x and y axis show herd name.

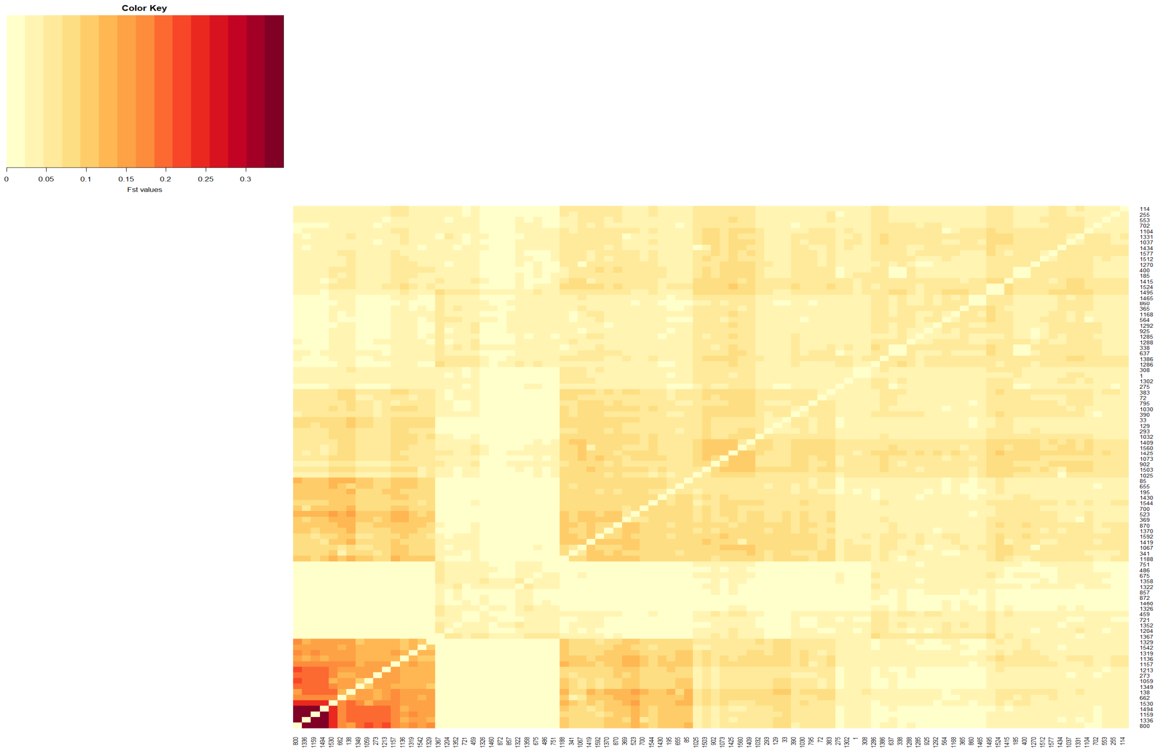

Figure S2. Heatmap based on pairwise *F*$_{ST}$ coefficients between the herds of Swallow-Belly Mangalica breed. The color key legend ranging from yellowish to dark red represents the scale of *F*$_{ST}$ coefficients from zero to onward. The x and y axis show herd name.

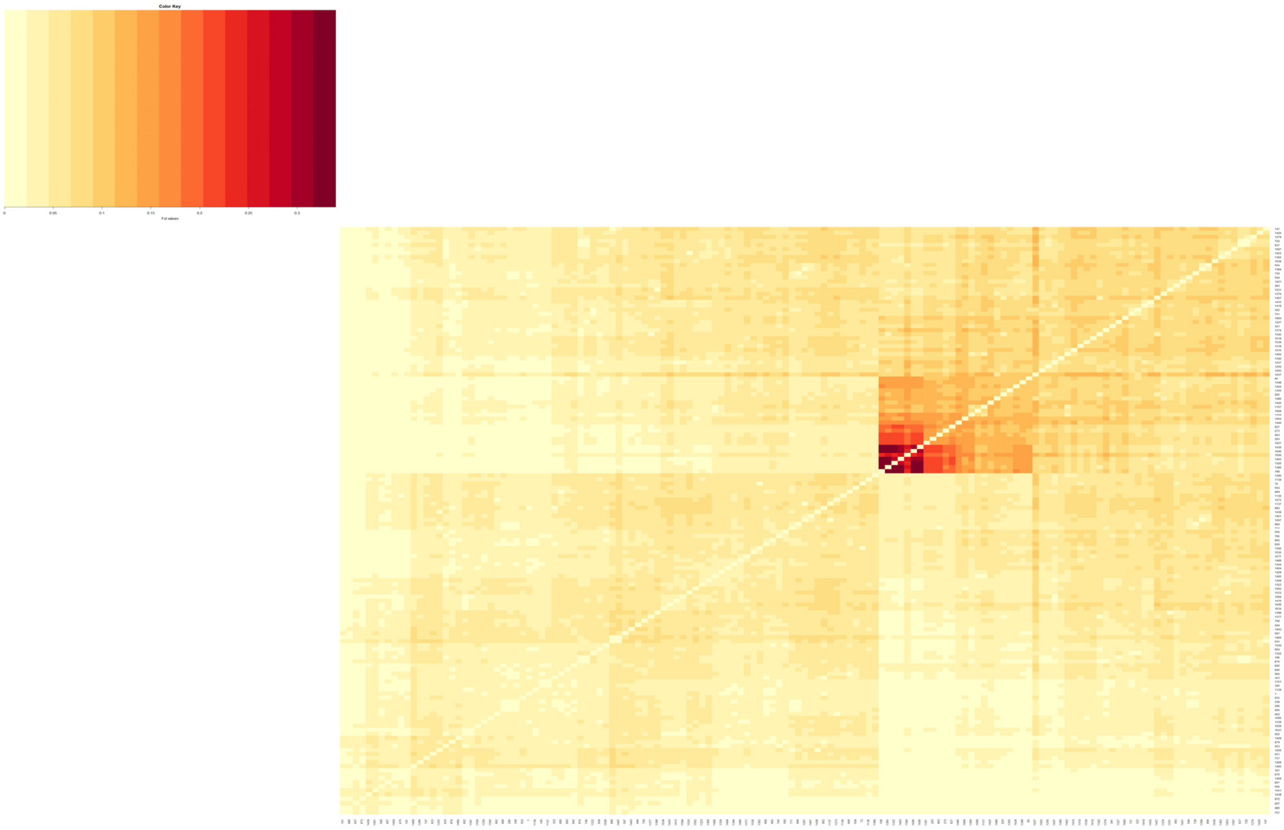

Figure S3. Heatmap based on pairwise F_{ST} coefficients between the herds of Red Mangalica breed. The color key legend ranging from yellowish to dark red represents the scale of F_{ST} coefficients from zero to onward. The x and y axis show herd name.

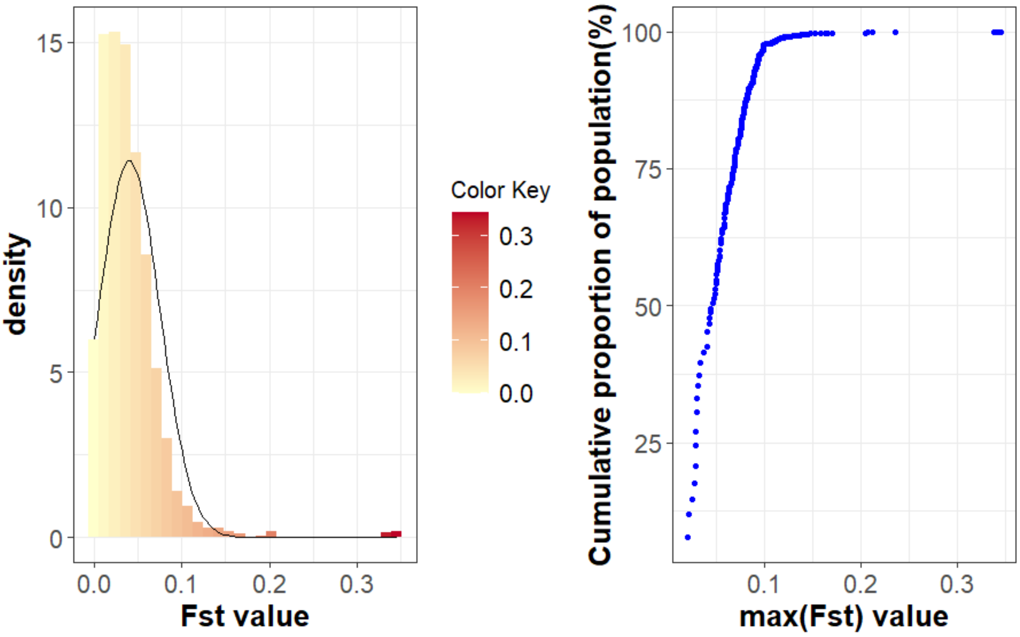

(a) **(b)**

Figure S4. F_{ST} coefficients in the Blonde Mangalica total herds: (a) Histogram of F_{ST} values with density; (b) Cumulative proportion of population related to the max F_{ST} of the herds.

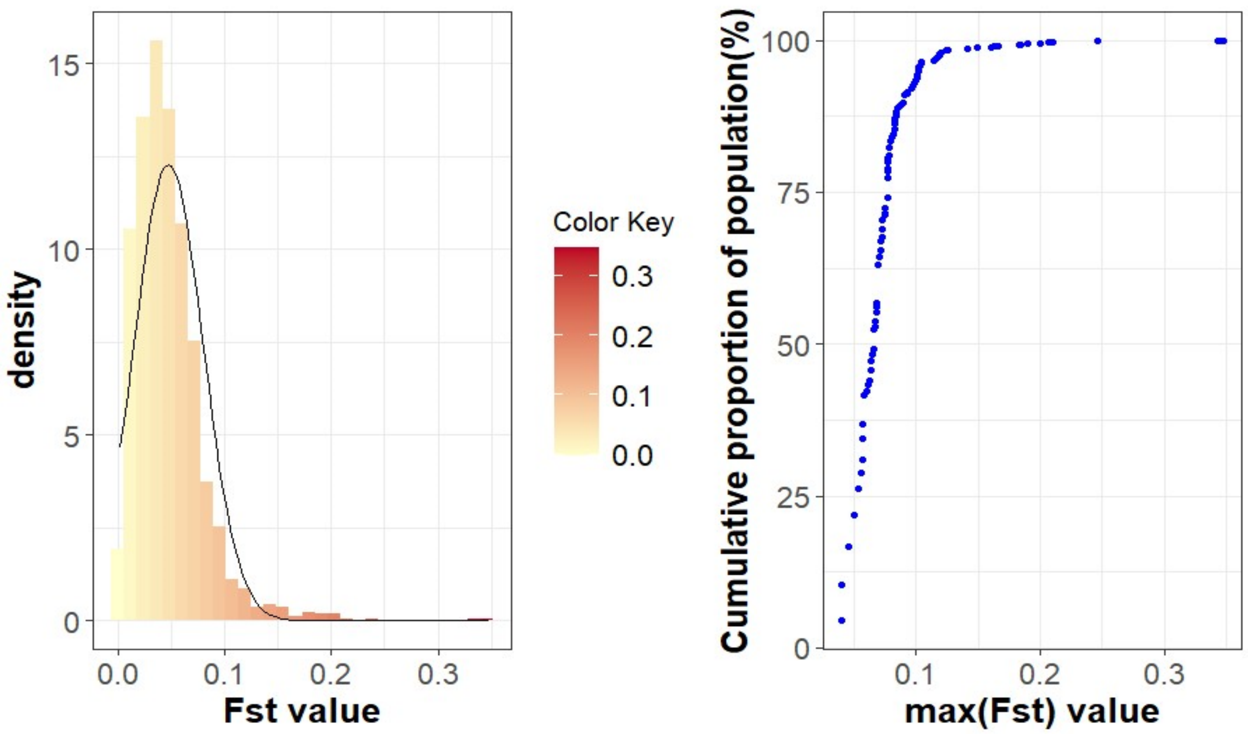

(a) **(b)**

Figure S5. F_{ST} coefficients in the Swallow-Belly Mangalica total herds: (a) Histogram of F_{ST} values with density; (b) Cumulative proportion of population related to the max F_{ST} of the herds.

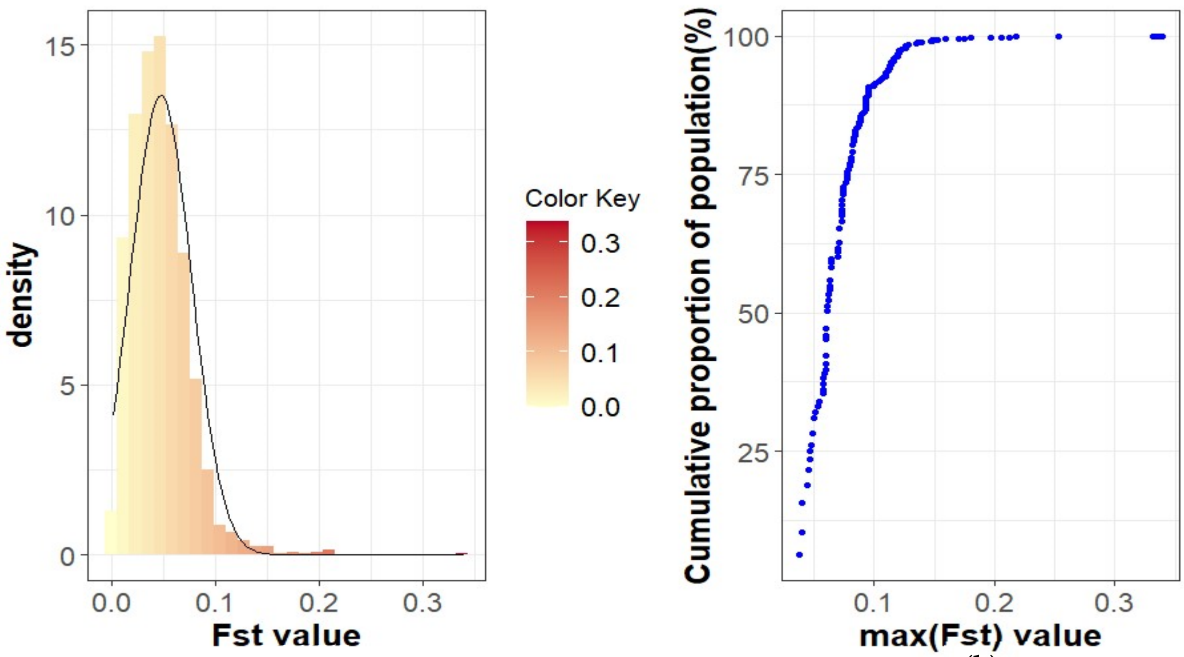

(a) **(b)**

Figure S6. F_{ST} coefficients in the Red Mangalica total herds: (a) Histogram of F_{ST} values with density; (b) Cumulative proportion of population related to the max F_{ST} of the herds.

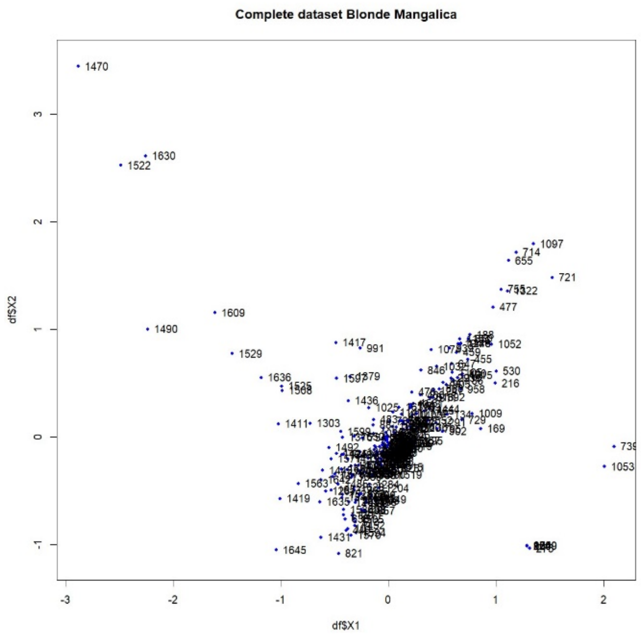

Figure S7. Multidimensional scaling (MDS) plot (MDS1&MDS2) of the Blonde Mangalica

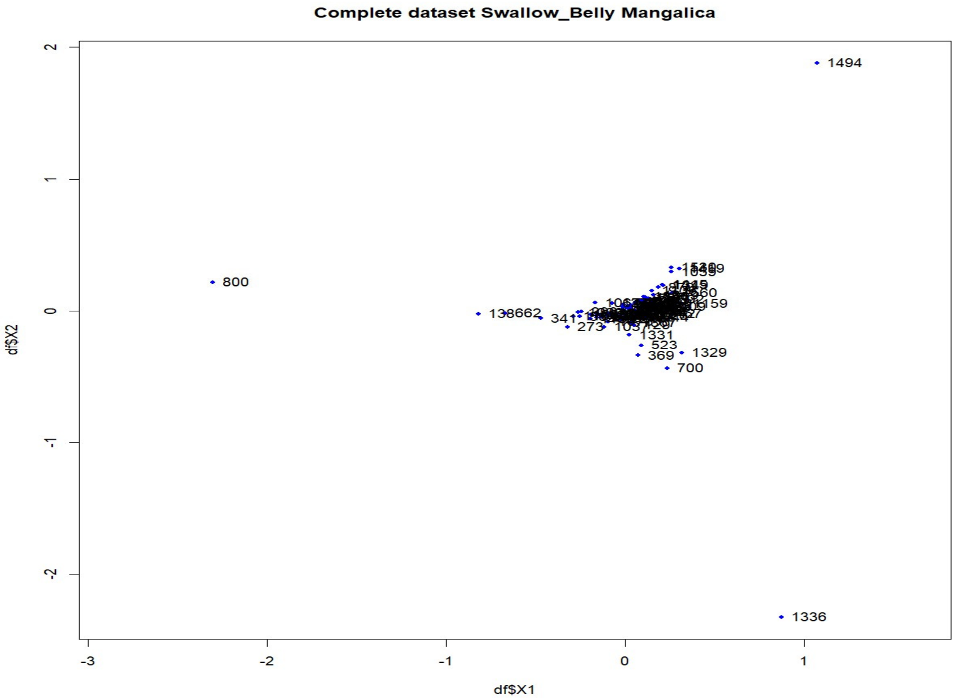

Figure S8. Multidimensional scaling (MDS) plot (MDS1&MDS2) of the Swallow-Belly Mangalica

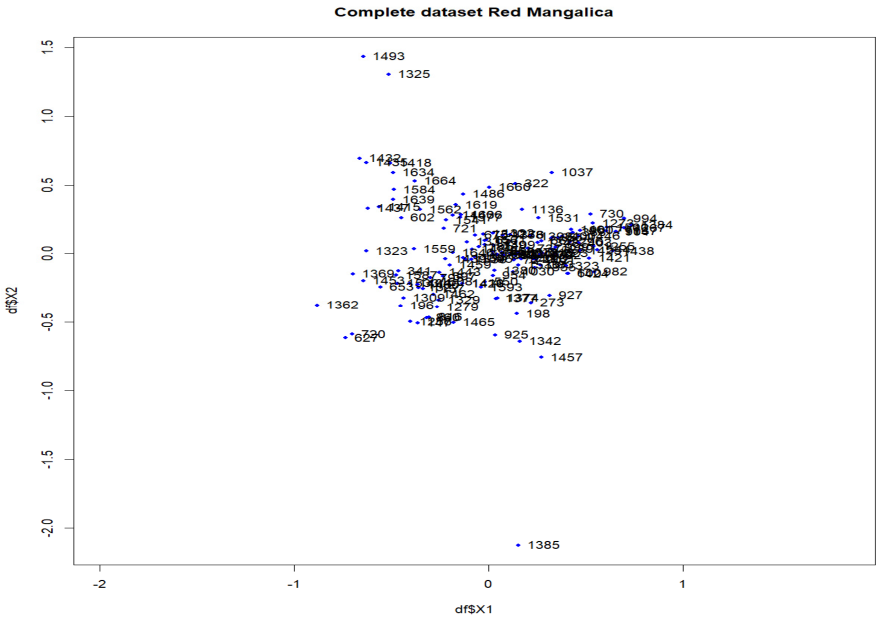

Figure S9. Multidimensional scaling (MDS) plot (MDS1&MDS2) of the Red Mangalica.

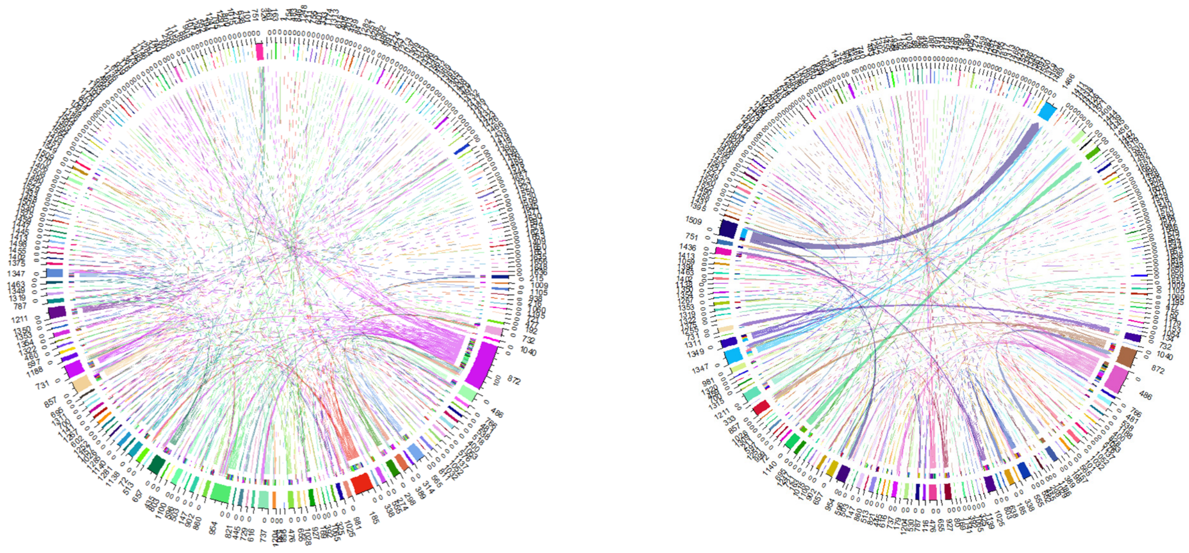

(a) **(b)**

Figure S10. Migration of the Blonde Mangalica in total herds: (a) Male; (b) Female.

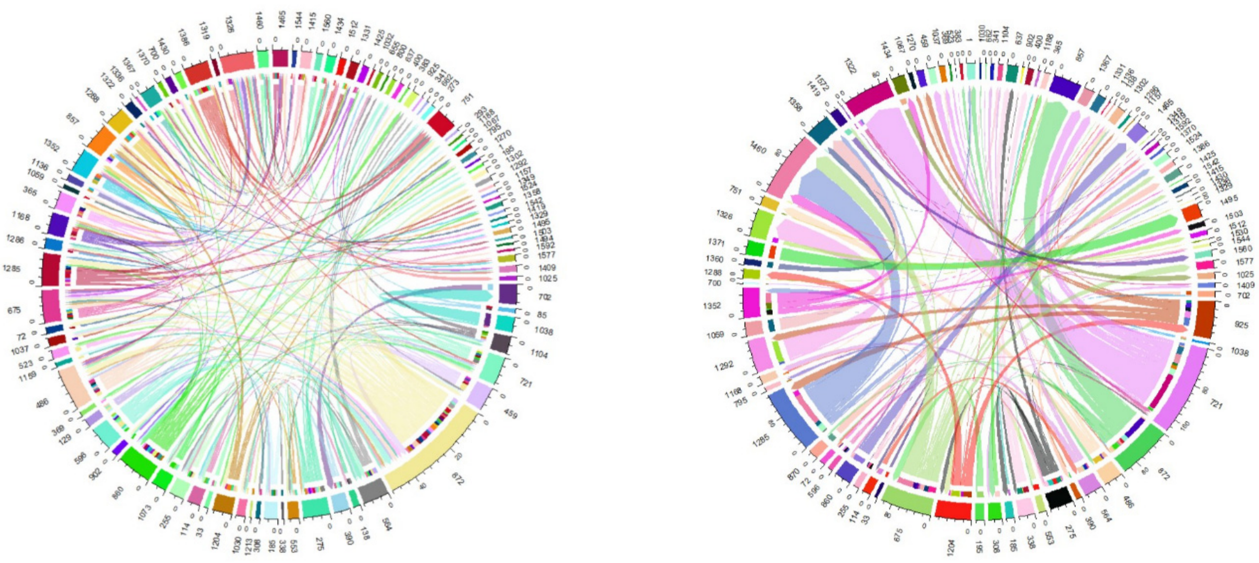

(a) **(b)**

Figure S11. Migration of the Swallow-Belly in total herds: (a) Male; (b) Female.

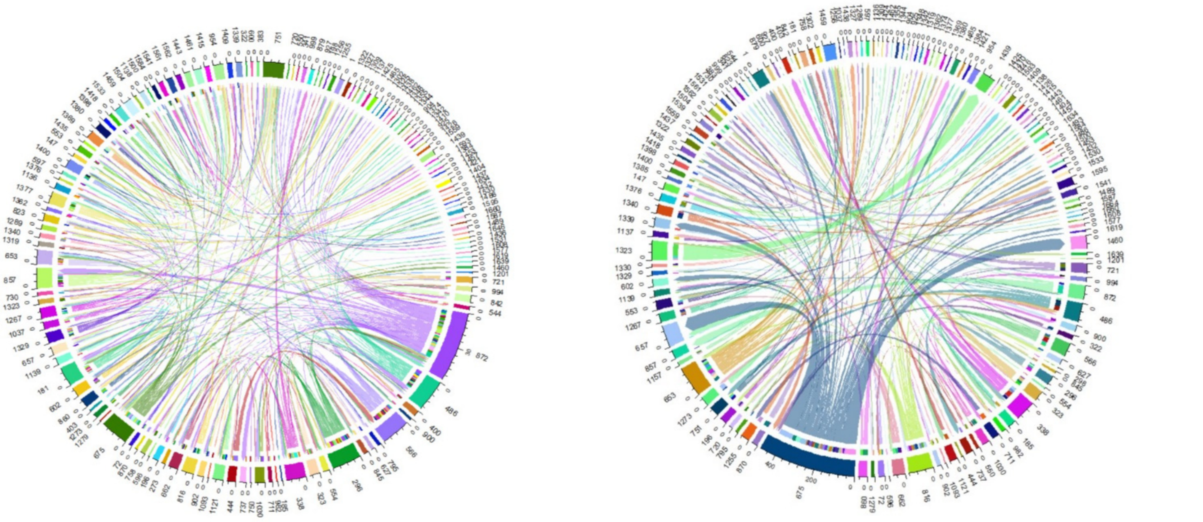

(a) **(b)**

Figure S12. Migration of the Red in total herds: (a) Male; (b) Female.
